# Evaluation of Cell Harvesting Techniques to Optimize Lipidomic Analysis from Human Meibomian Gland Epithelial Cells in Culture

**DOI:** 10.3390/ijms21093277

**Published:** 2020-05-06

**Authors:** Jillian F. Ziemanski, Jianzhong Chen, Kelly K. Nichols

**Affiliations:** School of Optometry, University of Alabama at Birmingham, 1716 University Blvd, Birmingham, AL 35294, USA; jfmead@uab.edu (J.F.Z.); jzchen@uab.edu (J.C.)

**Keywords:** human meibomian gland epithelial cells, meibomian gland dysfunction, mass spectrometry, cholesteryl esters

## Abstract

The lipidomic analysis of immortalized human meibomian gland epithelial cells (HMGECs) has been proposed as a preclinical model to study meibomian gland dysfunction. An in vitro study was conducted to evaluate neutral lipid recovery following three harvesting techniques and to identify candidate lipid biomarkers of HMGECs. HMGECs were cultured in serum-containing media for two days to promote lipid production. Cells were either harvested by 0.25% trypsin–ethylenediaminetetraacetic acid (EDTA), harvested by 10 mM EDTA, or simultaneously harvested and extracted by 2:1 chloroform–methanol (CM). After extraction by a modified Folch technique, the nonpolar phase was processed and infused into a TripleTOF 5600 mass spectrometer (Sciex, Framingham, MA, USA) with electrospray ionization. MS and MS/MS^all^ spectra were acquired. Nonpolar cholesteryl esters (CEs) were consistently detected in all samples, while wax esters were not. Only small differences in two out of twenty CEs were detected between harvesting methods. CM yielded less CE18:1 than the other methods but greater CE20:4 than the trypsin–EDTA method (*p* < 0.05 for all). Similar to human meibum, very long-chain CEs with carbon number (n_c_) ≥ 24 were detected in all samples and may serve as HMGEC lipid biomarkers. Further work is needed to address the absence of wax esters. Overall, the three harvesting methods are reasonably equivalent, though CM promotes much better efficiency and is recommended for higher throughput.

## 1. Introduction

Human meibomian gland epithelial cells (HMGECs) have been shown to accumulate lipids, respond to androgens, and express genes involved in lipogenesis and keratinization [1]. To date, however, there have been reports of the lipidome recovered from HMGECs differing from the typical lipid profile of normal human meibum [2,3]. Specifically, human meibum is low in phospholipids but high in long-chain wax and cholesteryl esters, while the HMGEC lipidome is the opposite [2,3,4,5,6].

One possible explanation for the inverse profiles between HMGECs and human meibum could be the use of an abrasive harvesting reagent that can cause increased cell permeability and the loss of intracellular contents. The combination of trypsin with ethylenediaminetetraacetic acid (EDTA) is a widely used harvesting technique in cell culture to promote the detachment of adherent cells from a growth surface. However, in metabolomic analyses, trypsin–EDTA treatment has been found to be too abrasive to the cell membrane, causing significant leakage of intracellular metabolites [7,8]. Neutral lipids produced by HMGECs are contained within intracellular vesicles [2] and therefore may be vulnerable to loss following trypsin–EDTA treatment.

Mass spectrometry is an analytical method that can be used to determine the composition of a complex fluid mixture, such as human tears, meibum, or HMGEC lysate. Mass spectrometry has been widely used in human tear and meibum analyses [4] but only in a minority of reports involving HMGECs [2,3]. The process of extracting lipids and injecting each sample into the instrument, however, is time consuming and costly. Therefore, an efficient harvesting method that limits the steps to extract lipids would expend less time and utilize fewer consumables, thereby enhancing research efficiency.

The primary objective of this pilot study was to assess trypsin-free harvesting techniques to optimize both the neutral lipid recovery from and analytical efficiency of HMGECs. In addition, considering that lipid production is the primary physiologic function of the meibomian gland, the identification of candidate lipid biomarkers that can be utilized to assess HMGEC function is paramount. Therefore, as a secondary objective, differences in nonpolar lipids between HMGECs and serum-containing media were evaluated and compared to the literature to determine their potential as HMGEC lipid biomarkers.

## 2. Results

**Figure 1:**
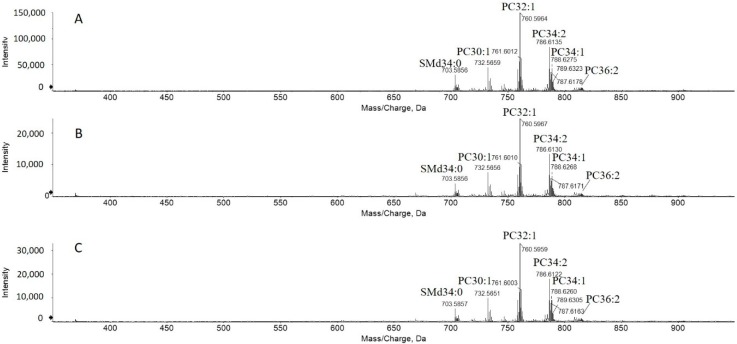
Representative mass spectra of lipids from human meibomian gland epithelial cells (HMGECs) acquired in positive ion mode. HMGECs were harvested by (**A**) 2:1 chloroform–methanol, (**B**) 10 mM ethylenediaminetetraacetic acid (EDTA), and (**C**) 0.25% trypsin–EDTA. Major peaks are identified. Labeling convention is carbon number in the acyl chains: double bond. Sphingomyelin with dihydroxy base (SMd), Phosphatidylcholine (PC).

The genotype of the HMGECs was confirmed by short-tandem repeat analyses. Our sample profile provided a 100 percent match to the reference profile at all sixteen loci [9]. Ultra-long chain cholesteryl esters (CEs) were detectable in all samples, thereby manifesting the expected phenotype of the glandular epithelial cells of the meibomian glands, as presented later. From all three techniques tested, successful harvesting and extraction were achieved (Figure 1) and, across techniques, the most abundant species were phosphatidylcholines (PC) and sphingomyelins (SM). In contrast, the peaks of wax esters and cholesteryl esters, common in human meibum, were limited in these MS spectra of HMGECs.

**Figure 2:**
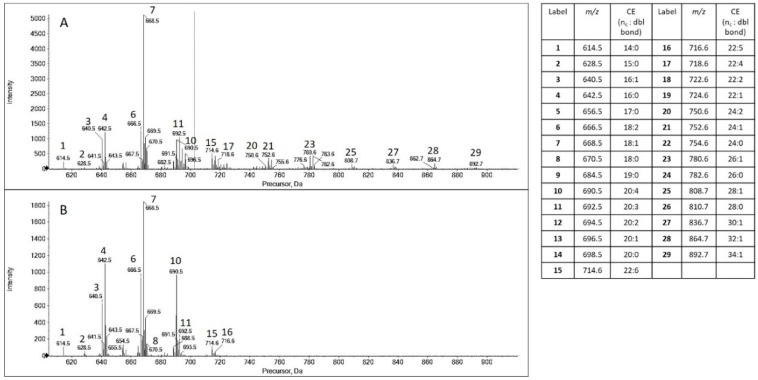
Representative pseudo precursor ion scanning mass spectra of cholesteryl esters (CEs, carbon number: double bond) extracted from MS/MS^all^ data for (**A**) HMGECs and (**B**) culture media. Only those CEs present in all samples are included in the legend. Only the most abundant peaks are labeled in the figures to aid readability. CEs with carbon number (*n*_c_ < 24) are present in both HMGECs and culture media, while CEs with *n*_c_ ≥ 24 are only present in HMGECs.

Considering that polar lipids, including phospholipids, are only a minor contributor to the lipid pool in human meibum [10,11,12], specific attention was given to the two most abundant classes: CEs and wax esters (WEs). Compared to MS analysis (Figure 1), the pseudo precursor ion scanning mass spectra extracted from MS/MS^all^ data are more sensitive to the detection of these nonpolar lipids [13]. Prior to lipid extraction, all media were aspirated, and the adhered HMGECs were washed twice to remove trace amounts of lipids remaining from the serum-containing culture media. Following analysis, CEs were consistently detected in all samples (Figure 2A). Similar CE diversity was present in cells and culture media when the carbon number (*n*_c_) was less than 24 (Figure 2A,B). However, very long- and ultra long-chain CEs with *n*_c_ ≥ 24 were only detected from cell samples and were absent from culture media (Figure 2A,B). WEs were not detected in any of the samples.

**Figure 3:**
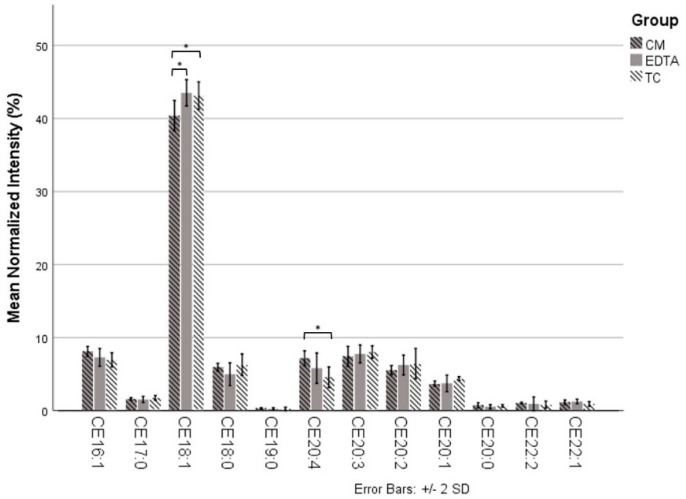
Comparison between the three harvesting techniques for the relative abundance of the cholesteryl esters (CEs) that were common (*n*_c_ < 24) to both cell and media samples. There were no differences between the three techniques for 10 of the 12 CEs. The chloroform–methanol (CM) method yielded less CE18:1 than the EDTA and trypsin-containing (TC) methods but greater CE20:4 than the TC method. * *p* < 0.05, chloroform–methanol (CM), ethylenediaminetetraacetic acid (EDTA), trypsin-containing (TC) (trypsin–EDTA).

**Figure 4:**
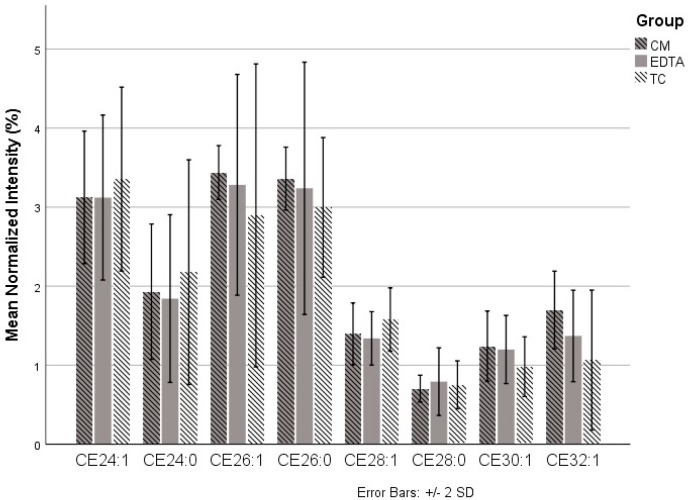
Comparison of the three techniques for the cholesteryl esters (CEs) that were only detected in HMGECs. Chloroform–methanol (CM), ethylenediaminetetraacetic acid (EDTA), trypsin-containing (TC) (trypsin–EDTA).

The relative abundance of each CE was compared between the three harvesting techniques, labeled as trypsin-containing (TC, 0.25% trypsin–EDTA), EDTA (10 mM EDTA), and chloroform–methanol (CM, 2:1 chloroform–methanol). No statistical differences were found between ten of the twelve CEs (Figure 3). The CM technique, however, yielded less CE18:1 (mean = 40.37) compared to EDTA (mean = 43.51, *p* = 0.02) and TC (mean = 43.14, *p* = 0.03) and greater CE20:4 (mean = 7.17) compared to TC (mean = 4.56, *p* = 0.02). There were no statistical differences between any of the longer-chain CEs (*n*_c_ ≥ 24) among the three different harvesting techniques (Figure 4).

## 3. Discussion

We sought to optimize neutral lipid recovery and analytical efficiency from HMGECs in culture, while simultaneously attempting to identify candidate lipid biomarkers. This pilot study suggests that CEs with *n*_c_ ≥ 24 may be biomarkers of serum-differentiated HMGECs. Previous reports have shown that ultra-long-chain CEs (*n*_c_ ≥ 26) are not detected in other epithelial cells [14] or in serum [15]. While the CE class, as a whole, is not specific to any particular tissue, CEs with ultra-long-chain fatty acids are very specific to meibomian and sebaceous glands [16]. CEs with *n*_c_ ≥ 24 are attractive candidates as lipid biomarkers, considering that very long-chain (*n*_c_ = 20 to 25) and ultra-long-chain CEs make up the most abundant CEs in normal human meibum [11]. Additionally, recent work by Borchman et al. [17] and Shrestha et al. [18] show that CEs (exact species not specified) are decreased in adults with meibomian gland dysfunction. Therefore, CEs with *n*_c_ ≥ 24—not found in culture media, serum, other epithelial cells, or other tissues (except sebaceous glands)—may have differentiating capacity among disease states, making them promising candidate biomarkers. Further validation of these CEs to assess for change in response to challenge and intervention is needed.

We also tested the hypothesis that trypsin-free harvesting techniques promote better neutral lipid recovery, presumably by maintaining the integrity of the cell membrane until the desired extraction step. All three methods used in this study are compatible with lipid extraction. Despite using the highly sensitive MS/MS^all^ technique, we were unable to detect WEs in any of the samples in this study, a surprising finding considering that WEs are a predominant component of the lipid pool in normal human meibum [11,19]. These results suggest that serum-differentiated HMGECs fail to produce measurable levels of WEs under our experimental conditions. Hampel et al. used similar conditions—serum-induced differentiation for a culture duration of one day or three days [3]. Although they were able to detect WEs, they reported that it was only 0.35 to 0.46 percent of the overall lipid pool. Sullivan et al. also reported a presence of WEs after serum-induced differentiation, and they maintained differentiating conditions for up to two weeks [2]. They, too, acknowledged the low detection of WEs, accounting for < 1 percent of the total lipid pool. Differences in experimental conditions and/or analytical strategies could account for the discrepancy in WEs between our study (not detected) and theirs (present but at low quantities). The decision to differentiate for two days in our experiments was based on several factors. Our main objective was to evaluate three different harvesting and extraction techniques, an objective that is largely independent of culture duration. Our second objective was to evaluate our data to identify potential candidate biomarkers of lipid-producing HMGECs. Hampel et al. reported that the nonpolar lipid content recovered from serum-differentiated HMGECs was greatest at one day and then was reduced over the following thirteen days [3]. Our pilot studies suggested that there were no qualitative differences in the overall lipid profile, including the classes of lipids produced, between 2, 9, 16, and 23 days of culture in serum-containing media [20]. Therefore, the decision to maintain differentiating conditions for two days was made in conjunction with both the literature and pilot studies, and the duration was sufficient to successfully address our aims.

Unlike WEs, very long-chain and ultra-long-chain CEs were consistently detected. There were only small differences in specific CE species between the different harvesting techniques. The CM method yielded less CE18:1 than both EDTA and TC and greater CE20:4 than TC. There were no differences in the remaining 18 CEs detected. The likelihood that these differences are attributable to the abrasive nature of trypsin, as originally hypothesized, is low, given that there were no differences between the milder EDTA technique and the more aggressive TC technique. CE18:1 is highly abundant in plasma (and presumably serum) [15], and both TC and EDTA methods require a second application of serum-containing media to prevent further protease and/or chelating activity. The chloroform–methanol in the CM method, however, is directly applied to the cells and simultaneously initiates the extraction process, thereby avoiding the second introduction of the serum lipidome. Therefore, the CM technique is not only more efficient, but it also reduces the opportunity for serum and media contamination. The difference in CE20:4 expression levels between the CM and TC techniques is potentially of interest, considering that it is present, albeit in low quantities, in normal meibum [13]. Further investigation is warranted.

In a similar manner to our previously stated rationale for maintaining differentiating conditions for two days, we chose to differentiate by serum alone because the differentiating agent had little influence on our primary objective (comparing harvesting and extraction techniques). However, the presence of serum, a rich lipid source, is capable of influencing the recovered lipidome either through contamination or through the induction of metabolic processes that can modify lipid production [21]. As already mentioned, the importance of careful washing to remove all traces of the serum lipidome cannot be understated. Furthermore, the CM technique helps to minimize opportunities for contamination by the serum lipidome. From a meibogenesis perspective, it is not possible to state to what extent serum lipids could have been internalized, processed, and ultimately expressed by the HMGECs in this study. The involved metabolic pathways are extremely diverse, highly complex, and still in the early stages of research [21]. In general, fatty acids—the building blocks of complex lipids—are believed to derive from intracellular acetyl CoA or from circulating fatty acids in the blood. The most abundant fatty acid in plasma is C18:1 (oleic acid) [15]. In this study, CE 18:1 was the most prominent CE, accounting for approximately 40 percent of all CEs. The source of oleic acid in CE 18:1 is purely speculative, but its high expression from HMGECs in this study, especially compared to its low expression in human meibum (approximately five percent [13]), could be a reflection of its uptake from serum. A similar pattern was observed for the cholesteryl ester of arachidonic acid (CE 20:4). Further research involving a serum-free environment is needed to better understand exactly how the meibomian gland synthesizes the different types of lipids and to what extent fatty acids are produced de novo versus taken up from the blood. Most importantly, CEs with *n*_c_ ≥ 24 are not present in serum-containing media. Therefore, these particular CEs are confidently derived from, or at least modified by, cultured HMGECs.

Importantly, across all of the different harvesting and extraction techniques, the lipidome from HMGECs still shows significant differences relative to the lipidome from normal human meibum. One possibility to explain these discrepant findings is that immortalized HMGECs may fail to fully differentiate in culture, as previously hypothesized by other groups [3,22,23], though the methodology of Schroder et al. [23] has recently been challenged [24]. Hampel et al. stated that HMGECs that are differentiated by serum reach only an early stage of differentiation [25]. Other groups, however, have reported successful differentiation through several mechanisms beyond serum [26,27,28,29,30,31]. These disagreements in the literature are likely the result of an ill-defined biomarker that is sensitive to the early, middle, and late stages of differentiation. Until comprehensive work can be done to define the stages of meibocyte differentiation on the molecular level, this issue will continue to be unresolved. In the meantime, the molecular analysis of meibum by mass spectrometry appears to be a specific approach for the quantitation of targeted meibum-relevant lipids. Our data suggest that cholesteryl esters, particularly those with *n*_c_ ≥ 24, are viable outcome measures for future experiments using HMGECs.

Another hypothesis to explain the discrepancies in the lipidomes is that HMGECs may be dependent on other glandular tissue or biochemical reactions in vivo to finalize meibum composition, an idea previously suggested by Chen et al. in 2010 [19]. It has been proposed, in dermal sebaceous glands, that phospholipid recycling occurs across the ductal epithelium [32]. This process may dramatically reduce phospholipid content in the final excreta. Therefore, it is possible that the HMGEC lipidome should not be identical to human meibum. Despite these differences, experimentation using HMGECs continues to be a powerful tool in dry-eye and meibomian gland dysfunction research. In vitro experimentation can answer research questions that are either poorly controlled or unethical to perform in human patients. To appropriately use preclinical models—such as HMGECs or another recently described mouse meibocyte cell line [33]—in experimental manipulations, researchers should use carefully optimized methodology and target parameters that have been validated as similar to human meibum, such as CEs with *n*_c_ ≥ 24. Further experimentation is needed to address the differences between HMGECs and human meibum, as well as to further validate responsive and physiologically meaningful outcome parameters.

In conclusion, we have demonstrated that CEs with *n*_c_ ≥ 24 are candidate lipid biomarkers of HMGECs. Further research is needed to validate these markers in other culture conditions that may influence HMGEC differentiation. The lipidome as determined by mass spectrometry, however, shows minimal variation when using trypsin-containing versus trypsin-free harvesting methods, particularly for these very long-chain and ultra-long-chain CEs. The CM technique, though, has the added advantage of improved efficiency as it reduces the need for reagent incubation times and several time-consuming washing steps. Reducing the duration of sample preparation makes the use of mass spectrometry, an already time-consuming method, more feasible and more accessible. When high output is needed, the direct application of chloroform–methanol to cells in culture serves as the preferred harvesting method, thereby improving research efficiency.

## 4. Materials and Methods

### 4.1. Cell Culture

Immortalized HMGECs were maintained in keratinocyte serum-free media (KSFM) supplemented with 5 ng/mL epidermal growth factor (EGF) and 50 µg/mL bovine pituitary extract (BPE) until 80% confluence [1]. To promote differentiation and lipid synthesis, the culture medium was changed to Dulbecco’s modified Eagle’s medium (DMEM) and Ham’s F12 (1:1) containing 10 ng/mL EGF and 10% fetal bovine serum (FBS) [2].

### 4.2. Harvesting and Lipid Extraction

After two days of culture, the culture medium was withdrawn and retained for lipidomic analysis. Three different harvesting techniques were evaluated. The first technique was the standard 0.25% trypsin–EDTA for 5 min at 37 °C. Following incubation, all cells were confirmed to have visibly detached from the growth surface. The second technique was 10 mM EDTA at 37 °C for a total of 10 min. Cells were monitored for complete detachment after 5 min, but complete detachment was not achieved until 10 min. The third technique represents a newer adaptation of the Folch extraction [34] that simultaneously harvests and extracts lipids from cells in culture. To avoid the common issue of plastic additives overshadowing the detection of neutral lipids by mass spectrometry, HMGECs were cultured in 6-cm glass petri dishes. At the time of extraction, the culture media was withdrawn and retained, and the cells were washed twice with ice-cold HPLC-grade water. A phosphate buffer was avoided to ensure compatibility with mass spectrometric analysis. Pre-mixed chloroform–methanol (2:1 *v*/*v*, 3 mL), prechilled to −20 °C, was directly applied to the glass petri dish surface with adhered HMGECs. The growth surfaces were then scraped with a stainless steel scraper, and the sample was transferred to a glass vial for the remainder of the extraction steps. All experiments were completed in triplicate.

Both cell lysates and culture media were analyzed. Lipids were extracted from all samples by a modified Folch technique [34]. Pre-mixed 2:1 chloroform–methanol (*v*/*v*) was added directly to each sample, followed by 50 mM ammonium acetate in HPLC-grade water for cells or 67 mM ammonium acetate for culture media. Consistent with the Folch technique, the final ratio of chloroform–methanol–water for all samples, both cells and media, was 8:4:3. The resulting emulsion was agitated at 350 rpm for 20 min on ice on an orbital shaker and then centrifuged at 1600× *g* for 5 min to promote stratification. The lower, nonpolar phase was withdrawn for subsequent mass spectrometric analysis. All steps involving organic solvents were performed with glass, stainless steel, or polytetrafluoroethylene (PTFE) and in the absence of any other plastics to minimize the interference of plasticizers.

### 4.3. Mass Spectrometric Analysis

All samples were then diluted tenfold with methanol and ammonium acetate (final concentration = 2 mM). The sample was directly infused into a Triple TOF 5600 mass spectrometer (Sciex, Framingham, MA, USA) with electrospray ionization at a flow rate of 7 µL/min, as previously described [13]. MS and MS/MS^all^ spectra were acquired in both positive and negative ion modes. MS/MS^all^ analysis was performed with the SWATH technology (Sequential Window Acquisition of All Theoretical mass spectra, Sciex), where product ion MS/MS spectra for all precursor ions were acquired in the *m*/*z* range of interest (200 to 1200) at every one Dalton step with 6 min for each detection mode. The collision energy was 40 eV for positive ion mode and −54 eV for negative ion mode. The collision energy spread was 40 eV for both modes. At least four rinses were performed between sample runs, and results were monitored by mass spectrometry to ensure that carryover was negligible.

### 4.4. Data Analysis

Up to 90 percent of normal human meibum is comprised of wax esters (WEs) and cholesteryl esters (CEs) [11,19]; therefore, these lipid classes were the primary focus of this pilot study. Only the lipid species that were present in all samples were included in the analysis. Signal intensity for each identified peak was normalized to sum intensity per class and was therefore reported as percent per class. Differences between harvesting methods were assessed by one-way ANOVA with Tukey post-hoc testing (SPSS v26, Armonk, NY, USA), when tests of normality (Shapiro–Wilk) and homogeneity of variance (Levene’s Test) were satisfied. In the few instances where these assumptions were violated, the nonparametric Kruskal–Wallis test was performed.

## Abbreviations

HMGEC	Human meibomian gland epithelial cells
CE	Cholesteryl ester
WE	Wax ester
PC	Phosphatidylcholine
SM	Sphingomyelin
EDTA	Ethylenediaminetetraacetic acid
CM	Chloroform–methanol
TC	Trypsin-containing
n_c_	Carbon number
KSFM	Keratinocyte serum free media
BPE	Bovine pituitary extract
DMEM	Dulbecco’s modified eagle’s medium
EGF	Epidermal growth factor
FBS	Fetal bovine serum
PTFE	Polytetrafluoroethylene
